# Lymphoma: New Diagnosis and Current Treatment Strategies

**DOI:** 10.3390/jcm11061701

**Published:** 2022-03-19

**Authors:** Kai Hübel

**Affiliations:** 1 Faculty of Medicine, University of Cologne, 50937 Cologne, Germany; kai.huebel@uni-koeln.de; 2 Department I of Internal Medicine, Center for Integrated Oncology Aachen Bonn Cologne Duesseldorf, University Hospital Cologne, 50937 Cologne, Germany

Historically, the treatment of patients with lymphoma has been based on three columns, with ascending importance: surgery, radiation, and chemotherapy. However, in recent years, the treatment landscape is rapidly changing. The improved descriptions of molecular processes inside the lymphoma cell and the disclosure of cell interactions with the microenvironment have made it possible to develop targeted therapies. These treatment approaches enable the direct or indirect elimination of lymphoma cells, but reduce the risk of side effects. Furthermore, the introduction of refined tools for diagnosis and the identification of molecular markers have increased the availability of precise therapy for the individual patient. The approaches of personalized and targeted treatment as a fourth therapeutic column are developing.

In this Special Issue, five excellent articles reflect this new area in the diagnosis and treatment of lymphomas, from different perspectives. Momotow et al. summarize the exciting treatment landscape in Hodgkin’s lymphoma (HL) [1]. The story of HL represents a remarkable development, from a fatal to a curable disease. Nowadays, more than 80% of patients with HL have a chance of being cured. This is mainly due to the definition of risk groups and the skillful combination of chemotherapies. Furthermore, the introduction of the anti-CD30 immunoconjugate Brentuximab Vedotin in patients with advanced classical HL improves not only the overall outcome, but also reduces the side effects of chemotherapy by omitting bleomycin [2]. Moreover, with the approval of the checkpoint-inhibitors nivolumab and pembrolizumab, novel treatment options have become available and have resulted in high response rates, even in chemotherapy-refractory and heavily pretreated HL patients. For example, in the Keynote-204 trial, pembrolizumab demonstrated significant efficacy in patients relapsing after autologous stem cell transplantation [3].

In patients with HL, the use of positron emission tomography (PET) offers a promising approach to reduce the number of chemotherapy cycles. Based on the results of the HD18 trial of the German Hodgkin Study Group, the current standard for patients in advanced stages under 60 years of age consists of four cycles of escalated BEACOPP if PET is negative after cycle two; otherwise, the patient will receive six full cycles [4]. Using this approach, most patients with advanced HL will be treated with just four cycles, thereby reducing toxicity without compromising efficacy.

The identification of patients with a lower risk profile during the treatment, as performed in the HD18 trial, was a considerable improvement in the treatment of HL. The identification of high-risk patients before start of treatment is still a particular challenge. In the Special Issue, Herraez et al. report a retrospective survey assessing the tumor burden though volume-based PET parameters instead of using the Ann Arbor classification technique [5]. They found that a higher metabolic tumor volume and total lesion glycolysis (TLG) were significantly associated with a higher incidence of stages III–IV, B-symptoms, hypoalbuminemia, and lymphopenia, and a higher international prognostic score. Furthermore, TLG was the best single PET/CT-related tumor-load parameter that significantly improved the assessment of risk. The value of these findings is limited by the retrospective design of the survey; however, if confirmed by larger prospective trials, these data may help to identify high-risk patients in advance and to plan appropriate therapies.

The use of PET/CT has increasingly become a standard diagnostic tool, not only for patients with HL, but also in patients with non-Hodgkin’s lymphoma (NHL). For example, in diffuse large B cell lymphoma, several studies have evaluated post-chemotherapy PET/CT, rather than disease bulk, to guide the application of radiotherapy [6]. However, PET/CT is not available at all centers, and is not refunded by health insurance in all countries. Thus, regular CT scans remain the standard of care for many lymphoma patients. The structured reporting of results is important for improving the communication between radiologists, members of multidisciplinary teams, and patients. The manuscript by Granata et al. builds structured CT-based reports (SRs) for lymphoma patients in the staging phase [7]. The authors used a modified Delphi process to develop SRs and to assess the levels of agreement. The SRs used standardized terminology and structures, in order to adhere to diagnostic and therapeutic recommendations and to facilitate enrollment in clinical trials.

The outcomes of patients with lymphoma are not only driven by the lymphoma itself, but also by the presence of other pathologies. Immune-mediated conditions, such as autoimmune diseases (AIDs), may be especially associated with the development of lymphoproliferative disorders. This is well described for chronic lymphocytic leukemia (CLL), in which autoimmune cytopenias, particularly autoimmune hemolytic anemia and immune thrombocytopenia, complicate up to 25% of cases [8]. Their occurrence is correlated with more aggressive disease. The association between AID and further lymphomas is less well understood, and the underlying mechanisms remain unclear. In the article by Masciopinto et al., the authors comprehensively review the current literature debating the role of AIDs in the prognosis of lymphoma [9]. They describe the molecular, genetic, and microenvironmental factors involved in the pathobiology of lymphomas. It is hypothesized that imbalances in the immune system are risk factors not only for the development of lymphoma, but also for poor prognoses. Moreover, the lymphoma itself may facilitate an impaired immune response, resulting in reduced treatment tolerability and worse outcomes. Further studies are needed to describe this correlation in more detail and to clarify whether lymphoma arising in the context of AID could constitute a distinct biological subtype.

Finally, the paper by Kholodnyk et al. focuses on the role of chemokine receptors CCR1 and CCR2 on mononuclear cells in CD38-positive CLL [10]. CCR1 and CCR2 may play a role in the development of inflammatory disorders, AIDs, and cancer progression [11]. The expression of CD38 in CLL is associated with an unmutated IGHV status, and therefore, with reduced overall survival [12]. In the presented trial in newly diagnosed CLL, positive correlations between the expression of CD38 and the expression of the chemokine receptors CCR1 and CCR2 on the peripheral blood CD19^+^CD5^+^ lymphocytes were found. This could indicate that the detection of CCR1 and CCR2 on circulating leukemic cells might be a reliable prognostic indicator in CLL. Furthermore, these receptors and their signaling pathways could be possible targets for the development of anti-progression therapeutics in CLL.

In summary, this Special Issue summarizes important recent advances in the diagnosis, treatment, and prognosis of patients with lymphoma, and opens perspectives to future clinical trials. As the Guest Editor, I express my gratitude to all the authors and reviewers, and to the journal team, for making this valuable Special Issue possible.
